# Altered volumetric and functional connectivity of the habenula in chronic insomnia disorder

**DOI:** 10.3389/fnins.2026.1794237

**Published:** 2026-04-10

**Authors:** Jingjing Sun, Kai Zhang, Panpan Li, Wenyue Xu, Kaimo Ding, Bidan Zhang, Bei Zhao, Danwei Zhang

**Affiliations:** 1 Zhenjiang Mental Health Center, Zhenjiang Fifth Affiliated Hospital of Jiangsu University, Zhenjiang, Jiangsu, China; 2 Zhenjiang Municipal Public Security Bureau, Zhenjiang, Jiangsu, China

**Keywords:** chronic insomnia disorder, fMRI, functional connectivity, habenula, Yeo-17 network

## Abstract

**Background:**

Previous findings have demonstrated that habenula (Hb) is a key hub for sleep regulation and emotion processing. However, its role in chronic insomnia disorder (CID) remains understudied. This study aimed to investigate the structural characteristics of the Hb and its functional connectivity (FC) with large-scale brain networks in CID patients.

**Methods:**

A total of 42 CID patients and 31 age-, gender-, and education-matched healthy controls (HC) completed clinical questionnaires, structural magnetic resonance imaging (sMRI) and resting-state fMRI (rs-fMRI).

**Results:**

No significant group differences were observed in the relative volumes or the laterality index between CID patients and HC. However, voxel-wise FC analysis showed increased FC between the left Hb and the right inferior frontal gyrus, right middle frontal gyrus, and left angular gyrus in CID patients, alongside decreased FC between the left Hb and left inferior frontal gyrus, left precentral gyrus, and right caudate nucleus. The right Hb exhibited decreased FC with the right middle cingulate cortex, supramarginal gyrus and postcentral gyrus. Region-of-interest (ROI)-wise analysis further demonstrated that the left Hb had significantly decreased connectivity with the posterior/anterior somatomotor networks (SM-d, SM-v) and posterior dorsal attention network (DAN-*p*) in CID patients.

**Conclusion:**

Our findings indicate that CID is characterized by asymmetric FC abnormalities of the bilateral Hb, particularly impaired connectivity between the left Hb and SM and DAN networks, without structural changes. These aberrations provide novel neuroimaging insights into the pathophysiology of CID and highlight the left Hb as a potential target for targeted interventions.

## 1 Introduction

Insomnia, the most prevalent sleep complaint worldwide, affects one-third of adults (Stein and Sareen, 2015). Epidemiological surveys indicate that 10%–20% of the global population experiences insomnia, with about half of these cases progressing to a chronic condition, namely chronic insomnia disorder (CID) (Buysse, 2013). From a public health perspective, CID substantially impairs patients’ quality of life and imposes a heavy socioeconomic burden (Dan-Dan et al., 2024; Damien and Bayon, 2010; Meagan et al., 2009). Pathologically, long-term insomnia is a recognized risk factor for various mental and physical disorders including anxiety, depression (Laura et al., 2022), hypertension (Sogol and Redline, 2017), and type 2 diabetes (Li et al., 2025).

Despite its considerable impact, the underlying neuropathological mechanisms of CID remain incompletely understood. In recent years, neuroimaging techniques have been widely employed to investigate neurobiological alterations and brain mechanisms underlying CID. These studies have confirmed that CID is associated with functional abnormalities in specific brain regions and networks, including dysfunction of the nucleus accumbens and medial prefrontal cortex circuit (Gong et al., 2021; Shao et al., 2020), enhanced thalamocortical connectivity related to sensory areas (Kim et al., 2021), altered connectivity patterns of suprachiasmatic nucleus (Yu et al., 2023), and an imbalance between the default mode network (DMN) and the frontal control network (Zheng et al., 2023).

As an evolutionarily conserved subcortical nucleus, the habenula (Hb) has been increasingly implicated in the pathophysiology of insomnia. On one hand, the Hb participates in regulating the sleep–wake cycle. Animal studies have demonstrated that manipulating glutamatergic neurons in the lateral Hb bidirectionally modulates both non-Rapid Eye Movement (NREM) and rapid eye movement (REM) sleep (Zhang et al., 2024). Furthermore, Hb lesions induce sleep fragmentation (Hsu et al., 2017). This nucleus exhibits an intrinsic circadian rhythm of neuronal discharge, regulating sleep by modulating melatonin Katherine et al., 2016 and catecholamine secretion (Salaberry and Mendoza, 2021). On the other hand, Hb is a key brain region for emotion regulation and reward processing. It encodes negative reward signals, mediates stress responses, and is closely related to common comorbidities of insomnia such as depression (Xin et al., 2025). Collectively, these findings suggest that the Hb dysfunction may play an important role in the pathogenesis of insomnia.

Recently, Dai et al. (Dai et al., 2025) reported that Hb volume is negatively correlated with sleep quality in healthy individuals. Moreover, a higher degree of left Hb lateralization was associated with more severe sleep disturbances, suggesting that Hb volume and lateralization may be involved in the basis of sleep regulation. Gong et al. (Liang et al., 2023) identified abnormal functional connectivity (FC) between Hb and the posterior cingulate cortex, a core node of the DMN. However, this finding that focuses on a single region may not be entirely representative of functional brain networks. The Yeo-17 network atlas (Thomas Yeo et al., 2011) can more precisely reflect the sub-region changes of different functional networks to enhance interpretability at the network level, but the existing studies have yet to conduct a systematic analysis between Hb and these sub-regions. Furthermore, there is still lack of exploration of the Hb structural volume in CID patients.

In this study, However, the existing studies have yet to conduct a systematic analysis between Hb and these sub-regions. Furthermore, there is still lack of exploration of the Hb structural volume in CID patients.

Therefore, this study aimed to address the aforementioned research gaps. Based on the Yeo-17 functional network atlas, and combined with structural magnetic resonance imaging (sMRI) and resting-state functional magnetic resonance imaging (rs-fMRI), we systematically characterized the structural features of the Hb in CID patients as well as its FC patterns with the 17 functional networks from a large-scale network perspective.

## 2 Subjects and methods

### 2.1 Subjects

A total of 77 subjects were recruited from October 2023 to September 2024 in Zhenjiang Mental Health Center, including 44 CID Patients and 33 age, gender, and education-matched healthy controls (HC). Three participants (two CID patients and one HC) were excluded due to abnormal signals in conventional T2 images. One control was excluded due to excessive head motion artifacts (exceeding 2 mm of maximal displacement and rotation). Ultimately, 42 CID patients and 31 HC were included in the study.

All participants were aged 18–65 years and right-handed. The CID patients met the diagnostic criteria of International Classification of Sleep Disorders, 3rd Edition (ICSD-3) for chronic insomnia disorders (Michael, 2014). Subjects in health control group had good sleep with Pittsburgh Sleep Quality Index (PSQI) score<7 (Buysse et al., 1989) and no history of use of sleep or psychiatric medications. The common exclusion criteria were as follows: (1) a history of neuropsychiatric disorders, brain surgery and trauma; (2) a history of alcohol, drug or psychoactive substance abuse; (3) contraindications to MRI; (4) pregnant or lactating women; (5) abnormal signals found in conventional MRI imaging.

This study was approved by the Medical Ethics Committee of Zhenjiang Mental Health Center. All the subjects agreed to participate in this study and signed informed consent prior to the study.

### 2.2 Clinical assessment

The PSQI was used to assess subjective sleep quality. The Self-rating Depression Scale (SDS) (Zung, 1965) and the Self-rating Anxiety Scale (SAS) (Zung, 1971) were used to assess depression and anxiety symptoms, respectively. All the assessment were completed by all subjects before MRI scan.

### 2.3 MRI data acquisition

sMRI data were acquired on a Philips 3.0-T MRI scanner (Philips, Best, the Netherlands) in Zhenjiang Mental Health Center. Before the scan, the subjects lay flat on the examination bed. Sponge earplugs were provided to each subject to reduce the discomfort caused by noise, and the head was fixed with a cover pad. All subjects were instructed to remain awake, with their eyes closed and head still, during the scanning process. Structural images were acquired using a T1-weighted three-dimensional magnetization intensity preparation fast gradient echo sequence (MPRAGE) scanning. Scanning parameters were as following: Pulse repeat time (repeat time, TR) = 2,300 ms, echo time (echo time, TE) = 2.95 ms, field of view (FOV) = 240 × 240 mm, matrix = 256 × 256, flip Angle = 12°, slice thickness = 1 mm, gap = 0 mm, number of slices = 160. rs-fMRI data were acquired from the gradient echo-echo planar imaging (GRE-EPI) sequence, with the following scanning parameters: 32 slices, TR = 2,500 ms, TE = 30 ms, FOV = 240 × 240 mm, matrix = 64 × 64, flip angle = 90°, slice thickness = 4 mm, number of slices = 32, number of time points, 240.

### 2.4 MRI data pre-processing

fMRI data underwent standardized preprocessing using the fMRIPrep pipeline (version 25.1.4) (Oscar et al., 2018). Specifically, (1) head motion correction; (2) slice-timing correction; (3) brain extraction using antsBrainExtraction in advanced normalization tools(ANTs); (4) spatial normalization to Montreal Neurological Institute (MNI)152NLin2009cAsym standard space (1 × 1 × 1 mm isotropic voxel) using ANTs; (5) smoothing (Gaussian kernel with 6 × 6 × 6 mm full-width at half maximum with Hb intentionally excluded from this step, notably, the Hb ROIs were masked out during spatial smoothing to ensure functional specificity; (6) temporal filtering (0.01–0.1 Hz) to retain low-frequency fluctuations relevant to resting-state FC; (7) nuisance signal regression (including white matter, cerebrospinal fluid, global signal, and head motion parameters). After the completion of data preprocessing, standardized images and brain masks of all subjects were visually inspected on a case-by-case basis to ensure the accuracy of spatial registration and the precision of the brain masks segmentation.

### 2.5 Hb segmentation and volumetry

To maximize the retention of individual anatomical geometric features, in this study, the volume of the Hb was measured within the native T1-weighted space of each subject. We adopted the high-resolution Human Connectome Project (HCP)-50 Hb spatial template based on the MNI152NLin2009cAsym standard space constructed by Joo-Won et al. (Joo-Won et al., 2016; Joo-Won et al., 2018). This template was constructed via myelin content-based segmentation of the human Hb at 3 T, with high reproducibility and anatomical specificity for the Hb (excluding adjacent thalamic and cerebrospinal fluid tissues). The template is publicly available at: https://github.com/junqianxulab/habenula_segmentation. Using the ANTs software (Version 24.2.06) and the spatial transformation matrix generated during the fMRIPrep preprocessing process, the left and right Hb probability maps were inversely transformed from MNI standard space to the individual T1w native space of each subject. Subsequently, a threshold of 0.1 was set for the probability maps in the native space, and the voxels above the threshold were retained as regions of interest (ROI). The absolute volumes of the left and right Hb (mm^3^) were then calculated by counting the number of voxels within the statistical mask. It should be noted that, due to the spatial resolution limitations of 3 T fMRI, the Hb ROIs extracted in this study represent the entire habenula complex, with no further subdivision of its internal subregions. The 0.1 threshold was selected to preserve the complete Hb structural morphology while minimizing interference from partial volume effects of surrounding thalamic tissue and ventricular margin cerebrospinal fluid.

To correct for the influence of individual head circumference differences on volume measurements, we used the whole brain mask generated by fMRIPrep to estimate the total intracranial volume (TIV), and divided the absolute volume by TIV to obtain the relative volume. We then obtained the relative volume of the left left Hb, the relative volume of the right Hb, and the total relative volume. Additionally, we calculated the laterality index (LI) using the formula $\mathrm{LI}=\frac{V_{\text{Left}}-V_{\text{Right}}}{V_{\text{Left}}+V_{\text{Right}}}$ to assess the asymmetry of the volume.

### 2.6 Resting-state FC analysis

#### 2.6.1 Seed definition

Based on the results obtained from the Hb space template segmentation in the previous part of the MNI152NLin2009cAsym standard space, the Hb template was first binarized with a probability threshold of 0.1 to generate individual Hb masks for all subjects (matching the segmentation criteria in the Hb Segmentation and Volumetry section). For each subject, the mean blood oxygen level-dependent (BOLD) time series of the left and right Hb seed regions were separately extracted from the preprocessed rs-fMRI data, with the time series of each seed region normalized to a zero mean and unit variance to eliminate amplitude differences across subjects. All time series extractions were implemented using Nilearn (v0.12.1) and FMRIB Software Library (FSL, v6.0) software packages.

#### 2.6.2 Voxel-wise FC

Voxel-wise whole-brain FC analysis was performed using Analysis of Functional NeuroImages (AFNI, v24.2.06) and R (v4.4.0). For each Hb seed region (left/right Hb), the Pearson correlation coefficients were calculated between the mean BOLD time series of the seed and the time series of every single voxel in the preprocessed whole-brain rs-fMRI data, generating a voxel-wise FC map for each subject and each seed region. To improve the normality of all FC maps, a Fisher’s r-to-z transformation was applied.

#### 2.6.3 ROI-wise FC

To explore the FC between the Hb and large-scale brain functional networks, we employed the Yeo 17-network parcellation atlas to divide the entire brain into specific sub-networks. This parcellation was selected for its high spatial resolution, excellent cross-dataset and scanner reproducibility, and comprehensive coverage of insomnia-relevant brain networks mediating arousal, motor function, attention, emotion, and self-referential processing. The atlas was first resampled to 1 × 1 × 1 mm isotropic voxel to match the spatial resolution of our preprocessed rs-fMRI data and then applied to parcellate the whole brain into 17 functionally distinct sub-networks. These networks include: Visual Network (Visual, VSL): VSL-1, VSL-2; Somatomotor Network (Somatomotor, SM): Posterior (SM-d), Anterior (SM-v); Dorsal Attention Network (Dorsal Attention, DAN): Anterior (DAN-a), Posterior (DAN-p); Ventral Attention Network (Ventral Attention, VAN): Anterior (VAN-a), Posterior (VAN-p); Limbic System (Limbic): Temporal lobe (LMB-t), Orbifrontal (LMB-o); Fronto-parietal Network (Fronto-parietal, FPN): Posterior (FPN-p), Posterior-inferior (FPN-d), Posterior-lateral (FPN-v); Default Mode Network (Default Mode, DMN): Temporal lobe (DMN-t), Posterior (DMN-p), DMN-1, DMN-2. The distribution areas of the networks in the brain are shown in Supplementary Figure S1.

For each subject, the mean BOLD time series of each of the 17 functional sub-networks was extracted using Nilearn (v0.12.1). Pearson correlation coefficients were then calculated between the mean time series of the left/right Hb seed regions and the mean time series of each sub-network, followed by Fisher’s r-to-z transformation to normalize the correlation values.

### 2.7 Statistical analysis

All statistical analyses and visualizations were conducted in R (Version 4.4.0) and AFNI (Version 24.2.06). The significance level for all statistical tests was set at *p* < 0.05 unless otherwise specified.

### 2.8 Demographic and clinical data

A two-sample t test was used to compare differences of demographic and clinical characteristics between CID and the HC groups. The chi-square test was used to investigate sex differences between CID and the HC groups.

### 2.9 Hb volumetric analysis

Given the distribution characteristics of the data, we used the non-parametric test method, the Mann–Whitney U test, to evaluate the inter-group differences in the absolute volume, relative volume, and LI of the left and right Hb between CID and the HC groups. The significance level for the inter-group comparison was set at *p* < 0.05.

#### 2.9.1 Voxel-wise FC analysis

To explore differences in voxel-wise FC between CID and the HC groups, the 3dttest++ module in AFNI was used to conduct a two-sample t test controlling for the effects of sex and age. To identify the statistically significant and spatially contiguous differences in the brain regions, we employed a method combining intensity threshold and cluster size to threshold the statistical results. The definition criterion for significant clustering were set as follows: the absolute *t*-value at the voxel level was greater than 2.5 ( ∣t∣>2.5 , two-tailed test), and the number of consecutive voxels within a cluster was at least 30.

#### 2.9.2 ROI-wise FC

To investigate FC between the Hb and predefined large-scale brain networks in CID and HC groups, independent samples t-tests were conducted to compare Fisher’s r-to-z transformed FC *z*-scores for each pair of left/right Hb and the 17 Yeo 17-network subnetworks. Given the multiple comparisons across functional subnetworks, the False Discovery Rate (FDR) correction (*q* < 0.05) was applied to control for Type I errors. Both uncorrected *p*-values and FDR-corrected q-values were reported for full transparency, with only results meeting the FDR-corrected threshold of *q* < 0.05 considered statistically significant.

## 3 Results

### 3.1 Demographic and clinical characteristics

Detailed clinical and demographic characteristics of participants are shown in Table 1. There were no significant differences in age, gender, or education level (*p* > 0.05) between the two groups. CID patients had significantly higher scores in PSQI, SDS, SAS than those in HC group (*p* < 0.05; Table 1).

**Table 1:** Demographic and clinical characteristics of the participants.

Variable	CID group (*n* = 42)	HC group (*n* = 31)	*t*/*χ*^2^	*p* value
Age (years)	46.12 ± 8.89	46.27 ± 7.71	−0.07	0.945
Gender			0.04	0.844
Female	24	17		
Male	18	14		
Education (yeasrs)	14.66 ± 3.35	14.23 ± 3.22	0.56	0.574
Duration (months)	24.33 ± 19.71	—	—	—
PSQI total score	14.57 ± 2.07	4.61 ± 1.08	24.36	<0.001
SDS score	55.35 ± 9.16	41.16 ± 4.49	7.94	<0.001
SAS score	57.95 ± 6.21	33.61 ± 4.23	18.81	<0.001

Clinical variables presented as mean and standard deviation.

CID, Chronic insomnia disorder; HC, healthy controls; PSQI, Pittsburgh sleep quality index; SAS, Self-rating anxiety scale; SDS, Self-rating depression scale.

### 3.2 Hb volumetric indices

**Figure 1:**
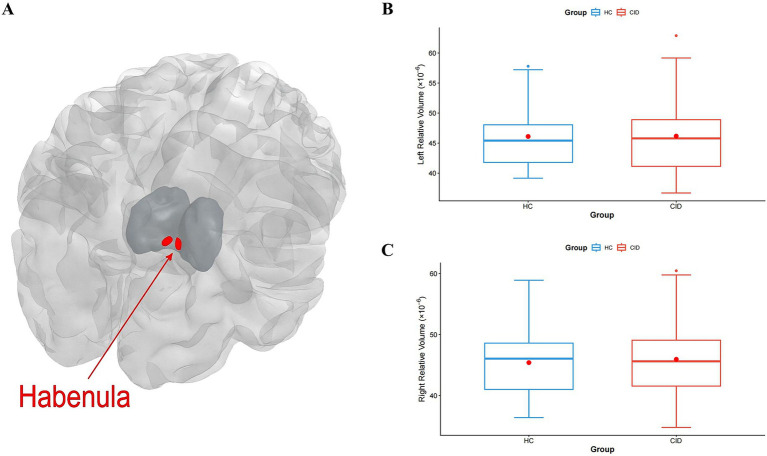
**(A)** A sketch map of bilateral Hb and comparisons of the **(B)** left Hb relative volume and **(C)** right Hb relative volume between CID and HC. CID: Chronic Insomnia Disorder; HC: healthy controls.

**Figure 2:**
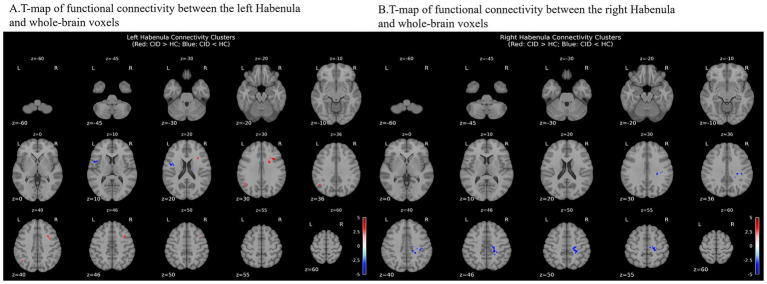
Voxel-wise functional connectivity differences in left Hb **(A)** and right Hb **(B)** between CID and HC group. Blue colors represent decreased connectivity, while red colors represent increased connectivity in the insomnia group. CID, Chronic insomnia disorder; HC, healthy controls.

Comparing the CID and HC groups, there was no significant difference in the relative volume of the left Hb (*p* = 0.918) and right Hb (*p* = 0.863). There was no significant difference in the total relative volume (*p* = 0.918) and LI (*p* = 0.686) between the two groups (Table 2, Figures 1, 2).

**Table 2:** Comparison of the volume and laterality index of the habenula between CID and HC groups.

Index	HC group	CID group	*p* value
Left relative volume	45.4236 (4.1795, 4.8051)	45.7905 (4.1144, 4.8902)	0.9176
Right relative volume	46.0503 (4.1003, 4.8591)	45.6096 (4.1553, 4.9074)	0.8628
Total relative volume	89.6468 (8.3062, 9.5892)	89.4798 (8.4289, 9.8021)	0.9176
Laterality index (LI)	0.0002 (−0.022, 0.0478)	0.0028 (−0.0224, 0.0268)	0.6859

*Values are presented as Median (Q1, Q3); **Group comparisons were performed using the Mann–Whitney U test. CID, Chronic insomnia disorder; HC, healthy controls.

### 3.3 Resting-state FC of the Hb differences

#### 3.3.1 Voxel-wise resting-state FC

**Figure 3:**
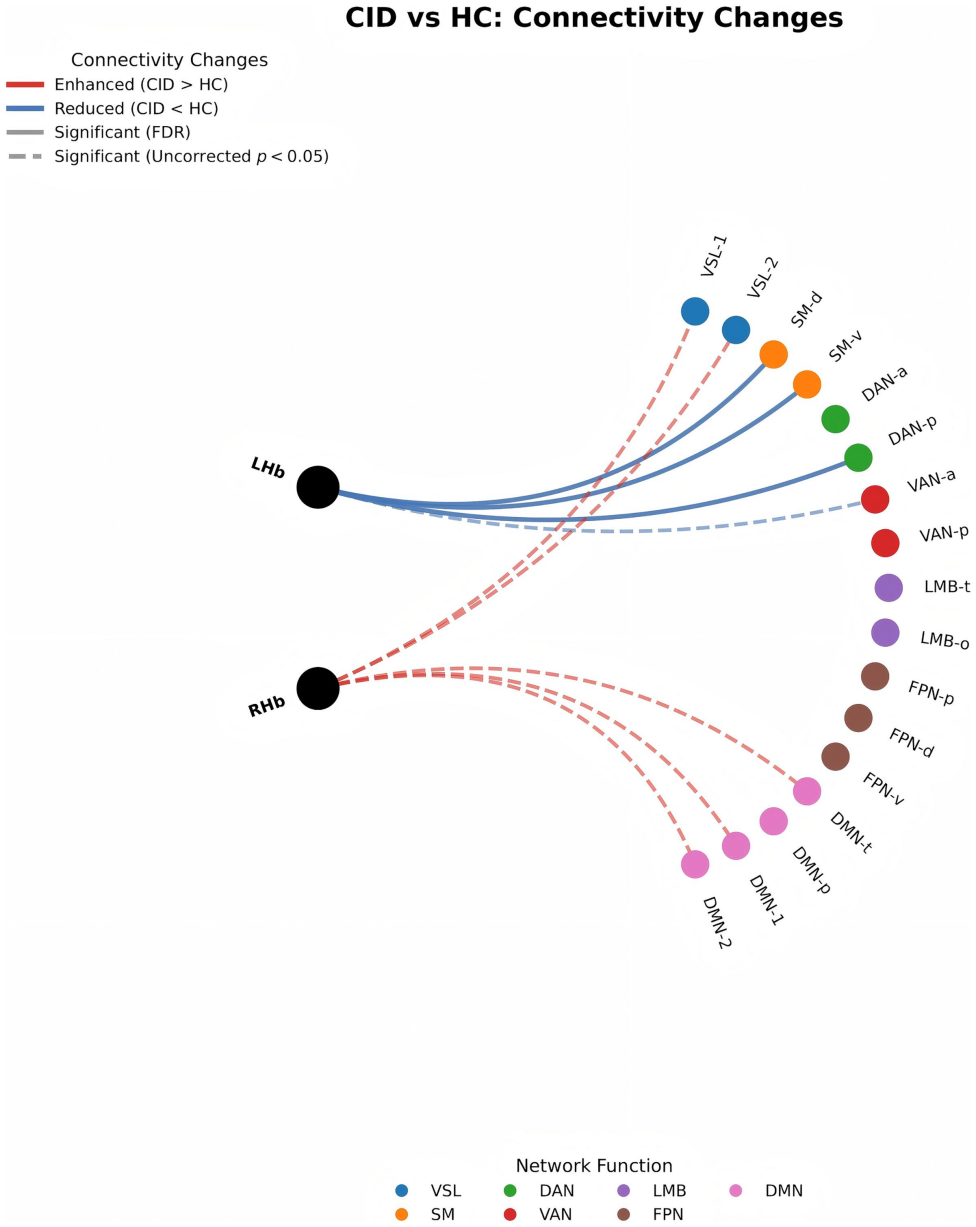
ROI functional connectivity differences of left and right Hb in the Yeo-17 subnetworks between CID and HC group. VSL, Visual; SM, Somatomotor; SM-d, Somatomotor Posterior; SM-v, Somatomotor Anterior; DAN, Dorsal Attention; DAN-a, Dorsal Attention Anterior; DAN-p, Dorsal Attention Posterior; VAN, Ventral Attention; VAN-a, Ventral Attention Anterior; VAN-p, Ventral Attention Posterior; LMB, Limbic; LMB-t, LimbicTemporal lobe, LMB-o, Limbic Orbifrontal; FPN, Fronto-parietal network; FPN-p, Fronto-parietal Posterior; FPN-d, Fronto-parietal Posterior-inferior; FPN-v, Fronto-parietal Posterior-lateral; DMN, Default Mode Network; DMN-t, Default Mode Temporal lobe; DMN-p, Default Mode Posterior (DMN-p); CID, Chronic Insomnia Disorder; HC, healthy controls; LHb, left habenula; RHb, right habenula.

The group differences in the bilateral Hb FC are illustrated in Table 3 and Figure 3. Compared with the HC group, the CID group showed significantly increased FC between the left Hb and several brain regions, including right inferior frontal gyrus (pars opercularis), right middle frontal gyrus, and left angular gyrus. Decreased FC was also found between left Hb and left inferior frontal gyrus (pars opercularis), left precentral gyrus, and right caudate nucleus (Table 3).

**Table 3:** Coordinates with significant differences functional connectivity of the habenula between CID and HC groups.

Brain region	BA	Voxel size	MNI coordinates (RAI)			Peak *t* value	Peak *p* value
			*x*	*y*	*z*		
Left Hb							
Right inferior frontal gyrus (pars opercularis)	9/44/6	121	36	12	33	4.988	<0.001
Right middle frontal gyrus	6	75	34	8	46	4.240	<0.001
Left inferior frontal gyrus (pars opercularis)	6/44	49	−50	6	10	−4.109	<0.001
Left precentral gyrus	6	45	−46	−3	23	−3.702	<0.001
Left angular gyrus	39	35	−48	−62	36	3.596	<0.001
Right caudate nucleus	–	33	14	−10	19	−4.109	<0.001
Right Hb							
Right middle cingulate cortex/paracentral lobule/precuneus	5/31	274	19	−35	47	−4.696	<0.001
Right supramarginal gyrus/postcentral gyrus	2/40	38	38	−28	34	−3.743	<0.001
Right postcentral gyrus	2/40	30	28	−31	38	−3.754	<0.001

BA, Brodmann area; MNI, Montreal Neurological Institute coordinates; Hb, habenula. *t* value represents the statistical value of the peak voxel. Positive values indicate that the connectivity strength of CID group is higher than that HC group, while negative values indicate that the connectivity strength of CID is lower than that HC group. The significance threshold is set at ∣t∣>2.5 , and the cluster size *k* ≥ 30.

For the right Hb, the CID group exhibited significantly decreased FC compared with the HC group in right middle cingulate cortex/paracentral lobule/precuneus, right supramarginal gyrus/postcentral gyrus, and right postcentral gyrus.

#### 3.3.2 ROI-wise FC with large-scale networks

As show in Table 4 and Figure 3, compared with the HC group, the FC was significantly decreased between left Hb and SM-d (*p* = 0.005), SM-v (*p* = 0.014), and DAN-p (*p* = 0.018) in CID group, while FC of the right Hb with all 17 sub-networks did not show statistically significant differences after FDR correction, although significant were observed in the VSL-1, VSL-2, DMN-t, DMN-1, and DMN-2 without FDR correction.

**Table 4:** ROI-wise functional connectivity differences between the bilateral habenula and Yeo-17 subnetworks in CID and HC groups.

Yeo-17 network	Left Hb Mean Z (HC /CID)	Left Hb Uncorrected *p*	Left Hb FDR-corrected *p*	Right Hb Mean Z (HC/CID)	Right Hb Uncorrected *p*	Right Hb FDR-corrected *p*
VSL-1	0.098/0.120	0.7366	0.8953	−0.135/0.004	0.0301	0.1508
VSL-2	0.146/0.132	0.8426	0.8953	−0.121/-0.03	0.0373	0.1508
SM-d	0.196/−0.019	0.0087	0.0493*	0.063/-0.029	0.3284	0.8959
SM-v	0.226/0.009	0.0028	0.0340*	−0.022/−0.031	0.9268	0.9268
DAN-a	0.161/0.143	0.7893	0.8953	−0.061/−0.034	0.7378	0.8959
DAN-p	0.231/0.034	0.0040	0.0340*	0.024/−0.049	0.3877	0.8959
VAN-a	0.160/0.048	0.0476	0.2023	0.025/−0.042	0.4533	0.8959
VAN-p	0.079/0.042	0.5862	0.8953	−0.037/−0.02	0.8275	0.9079
LMB-t	0.039/0.045	0.9289	0.9289	−0.052/−0.013	0.6439	0.8959
LMB-o	0.076/0.057	0.8150	0.8953	0.012/−0.048	0.4985	0.8959
FPN-p	0.081/0.022	0.3896	0.8953	−0.033/−0.062	0.6802	0.8959
FPN-d	0.106/0.064	0.4975	0.8953	−0.009/0.004	0.8545	0.9079
FPN-v	0.052/0.025	0.7024	0.8953	−0.037/−0.011	0.7097	0.8959
DMN-t	0.087/0.022	0.4673	0.8953	−0.131/−0.03	0.0443	0.1508
DMN-p	0.100/0.028	0.3284	0.8953	−0.114/−0.073	0.5991	0.8959
DMN-1	–0.005/0.028	0.5835	0.8953	−0.073/0.018	0.0388	0.1508
DMN-2	0.020/0.060	0.5902	0.8953	−0.095/0.034	0.0307	0.1508

CID, Chronic Insomnia Disorder; HC, healthy controls; Hb, habenula; VSL, Visual; SM-d, Somatomotor Posterior; SM-v, Somatomotor Anterior; DAN-a, Dorsal Attention Anterior; DAN-p, Dorsal Attention Posterior; VAN-a, Ventral Attention Anterior; VAN-p, Ventral Attention Posterior; LMB-t, LimbicTemporal lobe, LMB-o, Limbic Orbifrontal; FPN-p, Fronto-parietal Posterior; FPN-d, Fronto-parietal Posterior-inferior; FPN-v, Fronto-parietal Posterior-lateral; DMN-t, Default Mode Temporal lobe; DMN-p, Default Mode Posterior (DMN-p).

## 4 Discussion

This study systematically explored the structural characteristics of the Hb in CID patients and its FC with large-scale brain functional networks. The findings revealed that the relative volume and LI of the Hb in CID patients were not significantly different from those in HC, but there were abnormal FC between the Hb and multiple brain regions as well as specific large-scale functional networks, providing new neuroimaging evidence for elucidating the pathophysiological mechanism of CID.

Our findings showed no significant differences between CID and HC groups in the relative volumes of the left, right, and total Hb, nor in the LI, suggesting that chronic insomnia may not induce overt structural atrophy or hypertrophy of the Hb. This result is inconsistent with previous findings by Dai et al. (Dai et al., 2025) that Hb volume was negatively correlated with sleep disturbance in healthy individuals. Nevertheless, Luan et al. (Shu-Xin et al., 2019) reported no significant difference of absolute Hb volume was found in treatment-resistant depression. The discrepancy may be attributed to compensatory mechanisms during the chronic course of CID. Specifically, volume fluctuations may occur in the subclinical stage, and once the condition progresses to chronicity, these fluctuations are regulated by neural plasticity to reach a stable state, ultimately resulting in no significant volume differences. Alternatively, functional alterations may precede structural changes in CID, making FC a more sensitive marker in chronic stages.

As an evolutionarily conserved subcortical hub integrating emotional, motivational, negative reward signals, and sensory information (Jessica L et al., 2023; Hailan et al., 2020; Hyunchan and Hikosaka, 2022) and regulating sleep–wake cycles via melatonin and catecholamine secretion (Zhang et al., 2024; Salaberry and Mendoza, 2021; Katherine et al., 2016; Jorge, 2017), the Hb exhibits abnormal FC with multiple regions in CID, which is closely associated with the core pathological and clinical manifestations of the disorder. Abnormal hyperconnectivity between the Hb and core nodes of the FPN and DMN may reflect impaired suppression of cognitive hyperarousal, leading to persistent sleep-related rumination and excessive monitoring in CID patients (Harvey, 2002). In contrast, the decreased Hb connectivity with motor-related cortices constitutes a potential neural substrate for the physical fatigue commonly reported in CID (Seog et al., 2019), while the disrupted connection with the mesolimbic reward circuit may underlie the anhedonia and reduced motivation frequently comorbid with CID (Marjorie et al., 2021). Additionally, hypoconnectivity of the right Hb with regions responsible for salience monitoring and somatosensory integration reflects a breakdown in the integration of bodily signals with emotional and motivational states, which may be a compensatory adaptation to sustained hyperarousal or functional exhaustion of the Hb’s regulatory control over the salience network (Rita et al., 2022; Bedia et al., 2025), both of which are closely linked to the maintenance of insomnia symptoms.

The ROI-level analysis based on the Yeo-17 functional network atlas provided critical network-level specificity, with the reduced FC between left Hb and SM and DAN-p networks emerging as a key pathological feature of CID. SM mainly regulates the integration of somatic sensation and movement, while DAN is involved in goal-directed attention regulation (Michael and Raichle, 2007). Congruently, altered FC between SM and amygdala and striatum in CID patients has been reported in CID patients (Zhaoyang et al., 2011), and Hb-DAN disruptions have been linked to attentional bias in depression and schizophrenia (Rita et al., 2022; Hitoshi et al., 2021; Lei et al., 2018). The observed left Hb hypoconnectivity likely reflects impaired inhibitory control over motor and attention circuits, driving driving somatomotor hyperactivation and dorsal attention network dysfunction. Collectively, these alterations form a vicious cycle of somatic hypervigilance, attentional dysregulation, and persistent sleep disturbance. It is worth noting that this study did not find significant connection abnormalities between the right Hb and the 17 functional sub-networks. This may suggest that functional asymmetry of Hb in CID and the left Hb may play a more central role. Dai et al. (Dai et al., 2025) found that the degree of left Hb lateralization in healthy individuals was positively correlated with the severity of sleep disorders. Although this study did not find differences in the LI between groups, the specific abnormality in FC of the left Hb further supports that the functional asymmetry of Hb may be involved in the pathological process of sleep regulation.

Our findings, while preliminary, offer valuable translational insights for CID management aligning with advances in precision psychiatry that emphasize circuit-specific interventions. It is consistent with the growing role of rs-fMRI biomarkers in psychiatric biological subtyping and treatment guidance (Zhang et al., 2023; Jiaqi and Chen, 2025; Xing et al., 2024). First, the reduced FC patterns of left Hb connectivity could serve as candidate biomarkers for CID subtypes (Zhang et al., 2024). Second, the left Hb is a promising neuromodulation target. Clinical evidence shows that modulating Hb function via deep brain stimulation (DBS) modulates brain activities to improve sleep and mood (Wang et al., 2024; Jiang et al., 2024). Third, Hb FC profiles may guide personalized treatment. Reward dysfunction may benefit from Cognitive Behavioral Therapy for Insomnia (CBT-I), while somatic hypoconnectivity may respond to relaxation interventions.

Several limitations warrant consideration. First, the single-center cross-sectional design precludes us from establishing the causal relationship between the abnormal Hb function and the onset of CID, nor can it capture the dynamic changes in the structure and function of the Hb during the disease course. Second, the modest single-center sample may limit generalizability and detection of smaller effects. Third, information such as medication history, lifestyle and cognitive function of the patients was not collected, which may have potential confounding effects on the structure and function of the Hb (Zhang et al., 2025; Xing et al., 2026). Future large-scale, multi-center longitudinal studies are needed to validate our findings, and intervention studies are required to test whether Hb FC patterns can predict treatment response in CID patients. In addition, confirmatory studies directly contrasting the left and right Hb connectivity are needed to definitively establish lateralization.

In conclusion, this study demonstrated abnormal brain features of the bilateral Hb in CID patients as characterized by abnormal FC with multiple brain regions related to movement and attention, as well as the SM and DAN network. This study provides a new perspective for understanding the pathological of CID and offers potential imaging targets for the development of targeted intervention measures in the future.

## Data Availability

The raw data supporting the conclusions of this article will be made available by the authors, without undue reservation.
