# Supplementary figures and images for: Altered volumetric and functional connectivity of the habenula in chronic insomnia disorder
Source: Front Neurosci. 2026 Apr 10;20:1794237. doi: 10.3389/fnins.2026.1794237 (PMC13106473; doi:10.3389/fnins.2026.1794237)

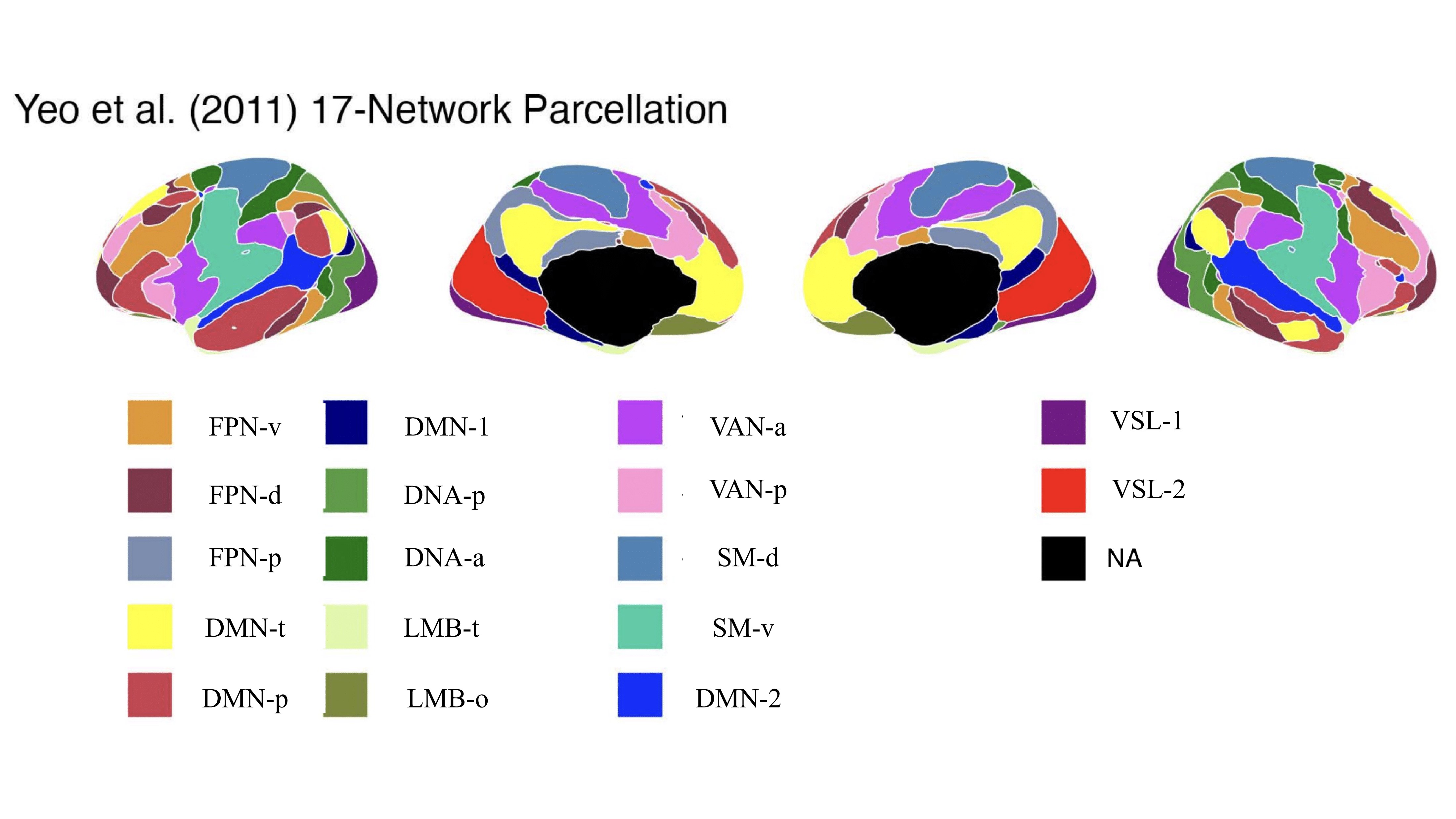

Supplement: Supplementary file 1 [file Image_1.JPEG]
